# Dengue and Chikungunya Virus Infections among Young Febrile Adults Evaluated for Acute HIV-1 Infection in Coastal Kenya

**DOI:** 10.1371/journal.pone.0167508

**Published:** 2016-12-12

**Authors:** Carolyne N. Ngoi, Matt A. Price, Barry Fields, Juma Bonventure, Caroline Ochieng, Grace Mwashigadi, Amin S. Hassan, Alexander N. Thiong’o, Murugi Micheni, Peter Mugo, Susan Graham, Eduard J. Sanders

**Affiliations:** 1 Centre for Geographic Medicine Research – Coast, Kenya Medical Research Institute (KEMRI), Kilifi, Kenya; 2 International AIDS Vaccine Initiative (IAVI) New York, New York, United States of America; 3 Department of Epidemiology and Biostatistics, University of California San Francisco, San Francisco, United States of America; 4 Division of Global Health Protection, Center for Global Health, Centers for Disease Control and Prevention (CDC), Nairobi, Kenya; 5 Kenya Medical Research Institute- KEMRI-CDC, Nairobi, Kenya; 6 Departments of Medicine, Global Health, and Epidemiology, University of Washington, Seattle, United States of America; 7 Nuffield Department of Medicine, University of Oxford, Headington, United Kingdom; University of California Davis, UNITED STATES

## Abstract

**Background:**

Fever is common among patients seeking care in sub-Saharan Africa (sSA), but causes other than malaria are rarely diagnosed. We assessed dengue and chikungunya virus infections among young febrile adults evaluated for acute HIV infection (AHI) and malaria in coastal Kenya.

**Methods:**

We tested plasma samples obtained in a cross-sectional study from febrile adult patients aged 18–35 years evaluated for AHI and malaria at urgent care seeking at seven health facilities in coastal Kenya in 2014–2015. Dengue virus (DENV) and chikungunya virus (CHIKV) were amplified using quantitative real-time reverse-transcription polymerase chain reaction. We conducted logistic regression analyses to determine independent predictors of dengue virus infection.

**Results:**

489 samples that were negative for both AHI and malaria were tested, of which 43 (8.8%, 95% confidence interval [CI]: 6.4–11.7) were positive for DENV infection. No participant was positive for CHIKV infection. DENV infections were associated with clinic visits in the rainy season (adjusted odds ratio (AOR) = 3.0, 95% CI: 1.3–6.5) and evaluation at a private health facility (AOR 5.2, 95% CI: 2.0–13.1) or research health facility (AOR = 25.6, 95% CI: 8.9–73.2) instead of a public health facility.

**Conclusion:**

A high prevalence of DENV infections was found in febrile young adult patients evaluated for AHI. Our data suggests that DENV, along with AHI and malaria, should be considered in the differential diagnosis of the adult patient seeking care for fever in coastal Kenya.

## Introduction

Fever is a common symptom among persons seeking healthcare in developing countries [1]. While various illnesses may prompt individuals to seek healthcare for fever, non-malarial febrile illnesses remain largely undiagnosed at many front-line health facilities due to a wide a range of potential aetiologies, overlapping clinical presentation and lack of diagnostic capacity [2]. With the World Health Organization’s (WHO) 2010 recommendation to confirm malaria infection by microscopy or by rapid diagnostic test (RDT) in patients presenting with a history of fever, the proportion of fevers that are recognized as likely due to diseases other than malaria is increasing [3].

There is little data on the aetiology of fevers in adults presenting for care in sub-Saharan Africa (sSA). In a systematic review of adults seeking outpatient care from clinics in sSA in the period 2003–2014, 34 (79%) of 43 studies identified focused on malaria only. The remaining studies investigated the following aetiologies: acute HIV-1 infection (AHI) and malaria (2 studies), human herpes virus-8 and hepatitis B virus (1 study), pneumococcal bacteraemia (1 study), flaviviruses (1 study), influenza viruses (1 study), Lassa virus (1 study), intestinal helminths (1 study) and schistosomiasis (1 study) [4].

Several seroprevalence studies have reported exposure to arbovirus infections including dengue virus (DENV) chikungunya virus (CHIKV) and O’Nyong Nyong (ONNV) infection in coastal Kenya [5–7]. Due to limited surveillance and lack of diagnostic capacity to screen for arboviral infections among patients seeking care, little is known on disease burden and transmission during interepidemic periods.

Dengue fever was first documented on the Kenyan coast during an outbreak in Malindi in 1982 [8], and more recently in Mombasa in 2013 [9] while chikungunya fever outbreaks were reported from Lamu and Mombasa in 2004 [10]. However, no assessment of DENV and CHIKV has been conducted outside of outbreaks among adult febrile patients seeking care in coastal Kenya. A recent study documented ongoing CHIKV transmission in Kwale County, south of Mombasa [7]. Laboratory diagnosis of CHIKV and DENV during acute phase of illness can be done through virus isolation or real-time reverse-transcription polymerase chain reaction (RT-PCR) [11–13]. In a study by Riswari et al., CHIKV viremia preceded fever onset by up to six days, and CHIKV quantitative (qRT-PCR) testing on serial specimens indicated that viremia lasted for 11–13 days [14]. DENV can be detected by RT-PCR two to ten days after onset of illness [15].

In 2013, a year of low malaria transmission in coastal Kenya, we conducted a pilot study on acute febrile illness and reported that 1.7% of young febrile adults who were seronegative or serodiscordant for HIV-1 had AHI and another 1.7% had malaria [16]. As less than 4% of fevers in pilot study participants enrolled in 2013 could be explained we set out to assess whether CHIKV or DENV were other causes of febrile illness in patients enrolled in the ongoing study.

## Methods

### Study setting

The study was conducted in two nearby towns in Kilifi County along the Kenyan coast: Mtwapa, which is situated 16 km north-east of Mombasa, and Kilifi, which is situated 42 km further north along the Mombasa-Malindi Road. The region experiences warm weather throughout the year with two rainy seasons, the long rainy season starting in March and lasting through June (4 months), and a shorter rainy period starting in October and continuing through December (3 months) [17]. The annual average temperature ranges between 26.5°C and 34.0°C (high) and 22.5°C and 24.5°C (low). The region has an average relative humidity of 60%.

### Study design

This was a cross-sectional study nested within an ongoing AHI study conducted between February 2014 and January 2015. Study volunteers were recruited from six health facilities in Mtwapa, including four private clinics, one research clinic operated by the Kenya Medical Research Institute, and one government health centre. In Kilifi, the Kilifi County Hospital was the only participating health facility. Study enrolments in Kilifi County hospital started mid-June 2014, at the end of the long rainy season.

As part of a standardized protocol, patients aged between 18 and 35 years who sought urgent health care at these facilities were screened for eligibility. Potentially eligible participants were assigned a risk score by summing points based on the following signs and symptoms and reported risk behaviour: one point each for generalized body pains or multiple partners in the past two months and two points each for documented fever (≥37.5°C axillary), reported diarrhoea, or symptoms compatible with a sexually transmitted infection including signs such as discharge or genital ulcers [16]. Inclusion criteria included age 18–35 years, a risk score of at least two, and willingness to be evaluated for acute or prevalent HIV-1 infection and, if febrile, for malaria. Patients with known HIV-1-seropositive status were excluded. Participants were also recruited for this study at five selected pharmacies in Mtwapa, and referred to any of the six health facilities for screening as described previously [16]. The Kiswahili name of the study was ‘*Tambua Mapema’*, which means “test early.”

### Study procedures

After obtaining written informed consent from eligible volunteers, a trained clinician from each respective facility collected demographic information, obtained a standardised clinical history including onset of illness, symptoms and signs present at health care seeking, and performed a targeted physical exam.

A 5-ml sample of venous blood was collected into EDTA. Onsite HIV-1 testing using two rapid diagnostic tests (RDT) Alere Determine^™^ HIV-1/2, (Alere Medical, Tokyo, Japan) and Uni-Gold^™^ (Trinity Biotech plc, Bray, Ireland) was done in parallel. Participants with documented fever were also tested for malaria using an RDT (Optimal, Flow Inc., Portland, Oregon, USA). All malaria-positive results were confirmed at the KEMRI-Wellcome Trust laboratories in Mtwapa and Kilifi using the same RDT test kit. Plasma was then separated and stored at -80°C in Kilifi, until further testing at the KEMRI-Centers for Disease Control and Prevention. Plasma was initially stored at a -20°C at the satellite laboratory in Mtwapa.

### Laboratory methods

In our study we used qRT-PCR to diagnose DENV or CHIKV infection. Automated Viral RNA extraction was done from 200 μL of plasma samples using the NucliSens easyMAG system (bioMerieux South Africa) according to the manufacturer’s instructions. Nucleic acid was recovered in 60 μL of elution buffer.

DENV was detected using the CDC DENV-1-4 real time RT-PCR assay kit for both diagnosis and identification of DENV serotypes using SuperScript^™^ III Platinum^®^ One-Step qRT-PCR system (Invitrogen, Carlsbad, California, USA) according to the manufacturer’s instructions and as previously described [15, 18].

CHIKV was detected using the AgPath-ID One-step RT-PCR Kit (Applied biosystems Carlsbad, California, USA) in a total reaction volume of 25 μL containing 5μL of RNA, 4.5 μL nuclease free water, 12.5 μL 2X RT-PCR buffer, 1μL of 25X RT-PCR enzyme mix, 0.8 μL of 20 picomoles forward (CHIK243–5′ GAY CCC GAC TCA ACC ATC CT 3′) and reverse primers (CHIK330–5′ CAT MGG GCA RAC GCA GTG GTA 3′) and 0.4 μL of 5 picomoles of the probe (CHIK273–5′ FAM-AG YGC GCC AGC AAG GAG GAK GAT GT-BHQ1 3′). Reactions were run in 96-well plates in the ABI 7500 FAST Dx thermocycler (Applied Biosystems, Foster city, California, USA) in the following cycling conditions: 45°C for 10 minutes, 95°C for 10 minutes, 45 cycles of 95°C for 15 seconds and 55°C for 1 minute.

### Data analysis

Overall prevalence was determined as a percentage of participants with detectable DENV and CHIKV infections over the total number of participants included. Exact 95% binomial confidence intervals (CIs) were calculated for prevalence estimates. Associations between binary or categorical variables were investigated using chi-square tests.

Bivariate logistic regression was used to determine associations with DENV infection (no CHIKV infections were observed). Variables significant at p ≤0.05 in bivariate analysis were entered into a multivariable model to identify independent associations with DENV. Crude and adjusted odds ratios (AOR) were calculated, with 95% confidence intervals (CI) and likelihood ratio test p-values. All analyses were done using STATA version 13.1 (StataCorp, College Station, Texas, USA).

### Ethical consideration

The study was approved by the KEMRI Scientific and Ethics Review Unit, and by the University of Oxford. All participants provided written informed consent for study participation.

## Results

### Characteristics of study participants

**Fig 1:**
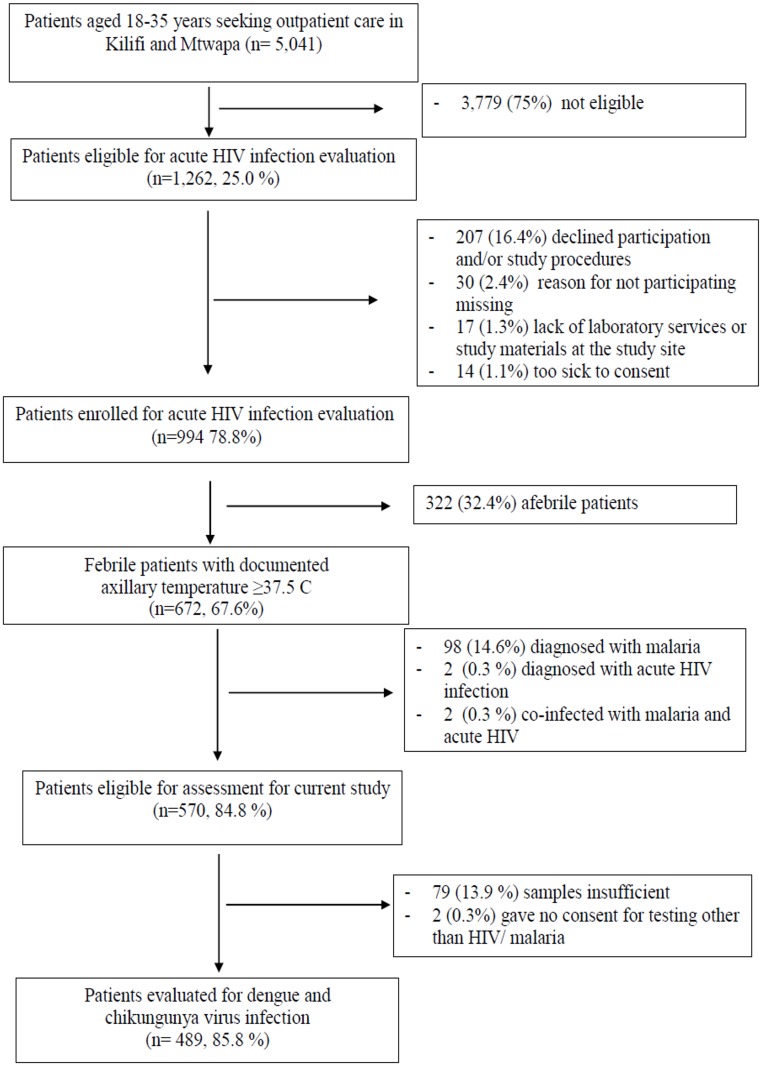
Flow chart of outpatients aged 18–35 years screened for evaluation at care seeking in Kilifi or Mtwapa, February 2014 –January 2015.

Among 5,041 participants aged 18–35 years seeking outpatient care, 994 were eligible and enrolled in the study, of whom 672 were febrile (Fig 1). We excluded 98 (14.6%) participants diagnosed with malaria, 2 (0.3%) participants diagnosed with AHI and 2 (0.3%) participants co-infected with malaria and AHI. Of the 570 remaining participants who were eligible for DENV and CHIKV assessment, 79 (13.9%) were excluded due to insufficient sample volume and 2 (0.3%) had not consented to testing for other causes of fever. In total, 489 participants were assessed for DENV and CHIKV infection (Fig 1).

Of the 489 participants included, 188 (38.4%) were men and 301 (61.6%) were women. Most were aged 18–24 years, married, and educated at the primary level or below. Half of the participants were unemployed, and about two thirds of all participants were enrolled in study sites from Mtwapa. Approximately 58% of the participants enrolled during the long and short rainy seasons. Almost half had first sought care elsewhere before visiting the study site, including one in six who had visited a pharmacy, while less than one in ten participants had a documented referral from a study pharmacy. About two thirds were evaluated at a government health facility, with the majority of individuals presenting within 7 days of onset of symptoms and reporting multiple symptoms (Table 1). The overall prevalence of newly diagnosed HIV-1 was 5.1% (3.2% in men and 6.3% in women, p = 0.1).

**Table 1:** Demographic and clinical characteristics of study participants (N = 489).

	Total (n = 489)	Male (n = 188)	Female (n = 301)
**Demographic characteristics**			
**Age group (years)**			
18–24	277 (56.6)	104 (55.3)	173 (57.5)
25–30	134 (27.4)	49 (26.1)	85 (28.2)
31–35	78 (16.0)	35 (18.6)	43 (14.3)
**Marital status**			
Single	224 (45.8)	126 (67.0)	98 (32.6)
Ever married	264 (54.0)	62 (33.0)	202 (67.1)
Missing	1 (0.2)	0 (0.0)	1 (0.3)
**Education**			
No or primary level education	269 (55.0)	70 (37.2)	199 (66.1)
Secondary or higher education	219 (44.8)	118 (62.8)	101 (33.6)
Missing education	1 (0.2)	0 (0.0)	1 (0.3)
**Employment status**			
Unemployed	247 (50.5)	54 (28.7)	193 (64.1)
Employed	242(49.5)	134(71.3)	108 (35.9)
**Residence**			
Mtwapa	335 (68.5)	153 (81.4)	182 (60.5)
Kilifi	154 (31.5)	35 (18.6)	119 (39.5)
**Seasonal variation**			
Dry season (January, February, July, August, September)	204 (41.7)	103 (54.8)	182 (60.5)
Rainy season (March through June, October through December)	285 (58.3)	85 (45.2)	119 (39.5)
**Health care seeking before study enrolment**			
Did not seek care elsewhere	267 (54.6)	94 (50.0)	173 (57.5)
Sought care elsewhere*	222 (45.4)	94 (50.0)	128 (42.5)
**Referred from a study pharmacy with a referral card**			
No	451 (92.2)	160 (85.1)	291 (96.7)
Yes	38 (7.8)	28 (14.9)	10 (3.3)
**Evaluated at**			
Government health facility or hospital	336 (68.7)	94 (50.0)	242 (80.4)
Private health facility	122 (25.0)	79 (42.0)	43 (14.3)
KEMRI research clinic	31 (6.3)	15 (8.0)	16 (5.3)
**Clinical presentation**			
**Reported duration of illness**			
Less than 7 days	423 (86.5)	159 (84.6)	264 (87.7)
More than 7 days	66 (13.5)	29 (15.4)	37 (12.3)
**Clinical presentation**			
Fever only	34 (6.9)	18 (9.6)	16 (5.3)
Fever and other symptoms and signs**	455 (93.1)	170 (90.4)	285 (94.7)
**HIV status**			
Negative	464 (94.9)	182 (96.8)	282 (93.7)
Positive	25 (5.1)	6 (3.2)	19 (6.3)

*Sought care elsewhere included: seeking care at a pharmacy (83), purchase of medication from a retail shop (73), having visited a different clinic other than the study site for the current illness (54), having visited the same clinic for the current illness (4), and use of medication prescribed during a previous illness (8).

**Other symptoms included: Headache (400), generalised myalgia (358), loss of appetite (245), sore throat (117), vomiting (61), diarrhoea (56), abnormal respiratory rate (33), conjunctivitis (6) and lymphadenopathy (50) (note: patients had multiple signs and symptoms).

### Prevalence of DENV and CHIKV

**Fig 2:**
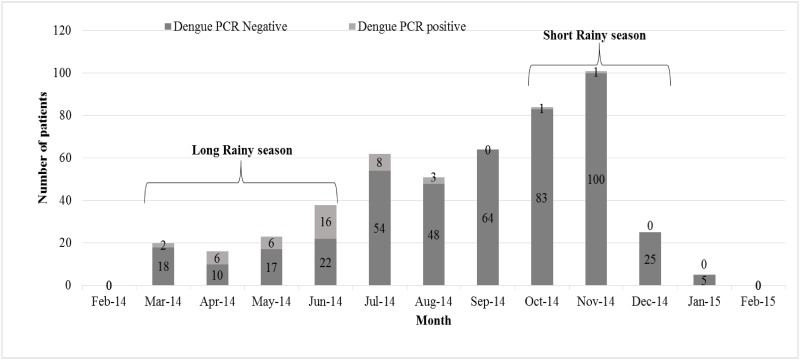
Study participants with and without dengue viral infection (N = 489).

None of the samples was positive for chikungunya virus infection. A total of 43 (8.8%, 95% CI: 6.4–11.7) participants tested positive for dengue virus infection. All samples were positive for DENV serotype 2, and one sample was also positive for serotype 3. Overall, the prevalence for DENV was 0% in Kilifi vs. 12.8% in Mtwapa (p = 0.001). However the prevalence of DENV infections among patients attending government facilities in Kilifi and Mtwapa was 0% (0/154) and 1.9% (3/159, p = 0.087) respectively (data not shown). The majority of dengue infections were observed during the long rains with peak transmission in June (Fig 2).

In bivariate analysis, DENV infections were more common among participants who were single, had a secondary education level or higher, were formally or self-employed, were referred from a study pharmacy or were seen at the KEMRI research clinic (Table 2).

**Table 2:** Bivariate and Multivariable analysis of demographic and clinical characteristics of febrile patients with dengue virus infection aged 18–35 years seeking care in Mtwapa or Kilifi, February 2014- January 2015 (N = 489).

	Acute dengue infection, n (%)	Bivariate analysis		Multivariable analysis	
		COR (95% CI)	*P* value	AOR (95% C.I)	*P-value*
**Demographic characteristics**					
**Gender**					
Male	20 (10.6)	Ref^1^			
Female	23 (7.6)	0.7 (0.4–1.3)	0.260	-	-
**Age group**					
18–24 years	27 (9.8)	Ref			
25–30 years	8 (6.0)	0.6 (0.3–1.3)			
31–35 years	8 (10.3)	1.1 (0.5–2.4)	0.370	-	-
**Marital status**					
Single	27 (12.1)	2.1 (1.1–4.1)	**0.020**	1.6 (0.8–3.4)	0.222
Ever married	16 (6.1)	Ref		Ref	
**Education level**					
Primary level education or less	14 (5.2)	Ref		Ref	
Secondary or higher education	29 (13.2)	2.8 (1.4–5.4)	**0.001**	1.3 (0.6–28)	0.586
**Employment status**					
Unemployed	15 (6.1)	Ref		Ref	
Employed	28 (11.6)	2.0 (1.1–3.9)	**0.031**	1.5 (0.7–3.2)	0.317
**Season of study visit**					
Dry season (January, February, July, August, September)	12 (5.9)	Ref		Ref	
Rainy season (March through June, October through December)	31 (10.9)	2.0 (1.0–3.9)	**0.050**	3.0 (1.3–6.5)	**0.004**
**Health seeking behaviour before visiting study sites**					
Did not seek care elsewhere	22 (8.2)	Ref			
Sought care elsewhere	21 (9.5)	1.2 (0.6–2.2)	0.636	-	-
**Referred from a study pharmacy with a referral card**					
No	32 (7.1)	Ref		Ref	
Yes	11 (29.0)	5.3 (2.4–11.7)	**< 0.001**	1.9 (0.7–4.8)	0.178
**Evaluated at**					
Government facility	9 (2.7)	Ref		Ref	
Private facility	20 (16.4)	7.1 (3.1–16.1)		5.2 (2.0–13.1)	
Research clinic	14 (45.2)	29.9 (11.3–78.8)	**< 0.001**	25.6 (8.9–73.2)	**< 0.001**
**Clinical presentation**					
Presenting with fever only	2 (5.9)	Ref		-	-
Presenting with fever and other symptoms^2^	41 (9.0)	1.6 (0.4–6.9)	0.512	-	-
**HIV status**					
HIV negative	42 (9.0)	Ref			
HIV positive	1 (4.0)	0.4 (0.1–3.1)	0.337	-	-

^**1**^ Reference

^**2**^ Other symptoms included: headache, generalised myalgia, loss of appetite, sore throat, vomiting, diarrhoea, respiratory rate, conjunctivitis and lymphadenopathy

In multivariable analysis, summarized in Table 2, independent predictors associated with DENV included presenting during the rainy season (AOR = 3.0, 95% CI: 1.3–6.5) and evaluation at a private health facility (AOR 5.2, 95% CI: 2.0–13.1) or the KEMRI research clinic (AOR = 25.6, 95% CI: 8.9–73.2), compared to a government facility.

**Fig 3:**
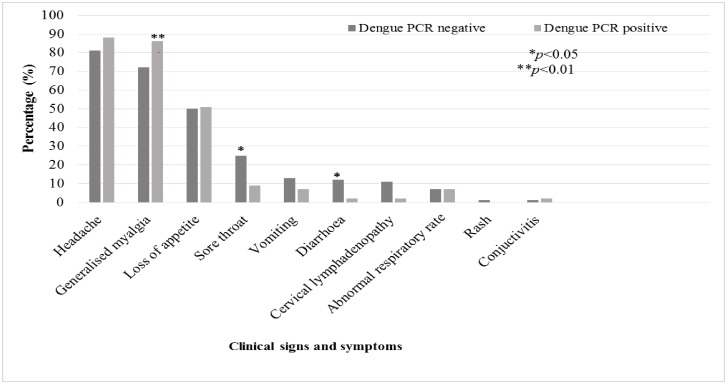
Clinical presentation of febrile patients aged 18–35 years seeking outpatient care in Mtwapa or Kilifi (February 2014- January 2015) with and without dengue virus infection (N = 489).

Participants with DENV were less likely to report sore throat (9.3% vs. 25.3%, p = 0.019) or diarrhoea (2.3% vs. 12.3%, p = 0.049) and more likely to report generalised myalgia (86.1% vs. 72.0%, p = 0.047), compared to participants without DENV (Fig 3). However, with only 43 DENV infections, the absolute number of symptoms or signs in DENV infected patients was small and clinical predictors were not included in the multivariable analysis.

## Discussion

We documented a substantial prevalence of DENV in young adult febrile patients seeking care at outpatient departments in coastal Kenya. While DENV was first reported in Kenya in the early 1980s during an epidemic in the coastal towns of Kilifi and Malindi [8], to our knowledge, this was the first study assessing the prevalence of DENV and associated factors among symptomatic patients seeking outpatient care outside of an epidemic.

All infections were identified in Mtwapa, a peri-urban town, located 16 km north-east of Mombasa, where an outbreak of DENV was reported in 2013 [9]. DENV infections identified in Mtwapa in 2014 likely reflect ongoing transmission following the DENV epidemic in Mombasa. Localised transmission of DENV during the rainy season is common as has been reported in Thailand and Gabon [19, 20], as dengue-transmitting *Aedes* mosquitoes are abundant during rains [21]. Surprisingly, participants with DENV were more likely to be diagnosed at private facilities or the KEMRI research clinic, which may reflect a selection bias, with symptomatic patients being more likely to seek care at these sites.

None of the participants tested positive for CHIKV. CHIKV infections have been reported in non-immune adults and children in Africa including Kenya [10, 22, 23]. We may not have detected CHIKV in our study population due to prior immunity in the young adult study population, or because CHIKV transmission may have been low in the study area in 2014. Similarly, we did not detect DENV among 154 febrile participants assessed in Kilifi, suggesting that DENV transmission may at times be localised. Our study did not assess for ONNV infection as we were not aware of ongoing ONNV transmission in Coastal Kenya at the time of planning the study [7]. Interestingly, in a follow up study characterising viral nucleic acids present in pooled plasma samples of 498 febrile patients through viral metagenomics (438 (88%) of plasma samples belonged to the present study), we detected DENV virus, but no CHIKV, or other alphaviruses (Ngoi et al, JVI, in press).

Our study highlights the importance of knowing the local differential diagnosis of febrile patients in resource-limited settings [2]. In addition to testing for malaria as recommended by the WHO, clinicians attending to febrile adult patients should provide HIV-1 testing, as febrile patients are more often HIV-infected [2, 16]. In facilities with the capacity for AHI testing [24, 25], clinicians should test patients who meet risk or symptom criteria for AHI [25, 26]. Although patients with DENV had more frequent generalised body pains, and less frequent sore throat or diarrhoea than patients without DENV, these characteristics were not helpful in identifying DENV among participants evaluated.

Our study had some limitations. First, we only included young adult patients evaluated within an AHI screening study at two sites in a small area of the Kenyan coast. Our study population from Kilifi only included patients reporting to a government hospital. We did not include patients reporting to private facilities or a research facility in Kilifi, nor did we recruit patients through pharmacies in Kilifi. Second, we excluded patients diagnosed with malaria or AHI; as we wanted to describe unknown causes of fever among non-malarial febrile illnesses. AHI and DENV coinfection has not been reported to our knowledge; concurrent DENV and malaria infection due *Plasmodium falciparum* or *plasmodium vivax* are rare [27], and both transmission vectors and transmission season are different [20]. Taken together, excluding patients with AHI or malaria represented approximately 15% of the study population and thus likely do not represent a significant bias. Third, we did not ask questions about factors previously associated with DENV, such as use of mosquito net or mosquito repellent, or recent travel outside Kenya [9]. Differences in these risk factors between participants recruited at our different study sites may have explained the different prevalence we found in Mtwapa.

Despite these limitations, we showed that DENV infections are an important cause of febrile illness among young adult patients seeking outpatient care in coastal Kenya. Ongoing surveillance for dengue viral infections and other arboviruses is recommended, and detection should lead to intensified effort to minimise mosquito breeding sites and promote personal protective measures. As causes of fever will largely remain undiagnosed without affordable and reliable point-of-care diagnostics, it is hoped that such tests will be developed in the foreseeable future. In conclusion, our study documented a relatively high prevalence of DENV in young febrile adults in Mtwapa. Dengue fever should be considered in the differential diagnosis of the febrile adult patient in Kenya.

## Supporting Information

S1 Dataset De-identified dataset. (CSV)

S1 Checklist STROBE checklist. (PDF)
